# Fluorescence Analysis of Quinine in Commercial Tonic Waters

**DOI:** 10.3390/mps8010005

**Published:** 2025-01-06

**Authors:** Artturi Harcher, Connor Ricard, Danielle Connolly, Isabel Gibbs, Jarve Shaw, Jillian Butler, Julia Perschbacher, Lindsay Replogle, Michaela Eide, Morgan Grissom, Oliver O’Neal, Quan Nguyen, Van Hac Nguyen, Michael Hunnicutt, Roaa Mahmoud, Soma Dhakal

**Affiliations:** Department of Chemistry, Virginia Commonwealth University, 1001 West Main Street, Richmond, VA 23284, USA

**Keywords:** quinine, fluorescence spectroscopy, quantitative analysis, detection, food and drug chemistry

## Abstract

Quinine is known for treating malaria, muscle cramps, and, more recently, has been used as an additive in tonic water due to its bitter taste. However, it was shown that excessive consumption of quinine can have severe side effects on health. In this work, we utilized fluorescence spectroscopy to measure the concentration of quinine in commercial tonic water samples. An external standard method was used to calculate the concentrations of quinine in two commercially available tonic water brands, namely Canada Dry and Schweppes, and compare them to the maximum allowable concentration of quinine in beverages. Upon analysis of the data collected by five different groups, the levels of quinine were found to be above the average concentration in most commercial tonic water samples, but below the maximum permitted concentration. Moreover, the five replicate sets of data demonstrated high reproducibility of the method employed in this study. The simple yet instructive protocol that we developed can be adapted to determine the concentration of other fluorescent compounds in foods and beverages. Further, the presented method and detailed protocol can be easily adopted for undergraduate labs and in chemical education.

## 1. Introduction

Quinine is historically known as an antimalarial drug used to treat the mosquito-borne disease malaria [1]. Due to its bitter taste, it is also used as a flavoring agent in soft drinks, such as tonic water [2]. However, despite its therapeutic applications, the U.S. Food and Drug Administration (FDA) has limited the maximum allowable amount of quinine in tonic water to 83 ppm [3] due to the adverse effects that quinine can cause if consumed in high doses, such as nausea, vomiting, kidney injury, and disseminated intravascular coagulation [4,5,6]. Therefore, it is important to employ analytical methods to check the levels of quinine in food products and beverages to ensure they meet regulatory requirements. This work serves as a good example of how to monitor fluorescent analytes in drinks. Structurally, quinine is characterized as a fluorescent molecule with analytically useful excitation wavelengths at 250 and 350 nm and a fluorescence emission wavelength at 450 nm in dilute acidic solutions [4,7]. Compared to other fluorescent molecules examined, such as pyrene in hexane and 2-acetylnaphthalene in water, quinine is fairly stable in acidic solutions and is not highly susceptible to quenching by oxygen [8]. Moreover, the ease of handling and straightforward sample preparation of quinine makes its analysis more feasible under a relatively safe laboratory environment.

Several instrumental techniques have been reported for the detection and determination of quinine in biological and food samples. For example, Castro et al. developed a fourth-order derivative spectrophotometric method to determine quinine in soft drinks in the presence of other additives [9]. Other studies used atomic absorption spectrometry [10], flow-injection chemiluminescence [11,12], gas chromatography [13], and liquid chromatography [14,15]. Although these methods are sensitive and eliminate interference from the sample matrix, they are generally time-consuming and require complicated sample preparation steps and data analysis procedures. Therefore, in this work, we developed a simplified and detailed experimental protocol using a widely employed and known technique, fluorescence spectroscopy, to determine quinine concentration in carbonated beverages (tonic water samples). A few studies have been reported in the literature for the detection of quinine using fluorescence-based assays [16,17,18,19]. For example, de Souza et al. devised an image-based fluorometric system equipped with an LED camera and a digital microscope to detect quinine in commercial samples [18]. Although high quantification limits were achievable using this fluorometric assay, the proposed method is relatively complex involving the use of mathematical models and post-analysis software for data acquisition and processing. Similarly, while Tsaftari and coworkers presented a paper-based fluorometric technique requiring minimal use of instrumentation for the rapid detection of quinine [19], the paper-based platforms are typically fragile and require careful handling to ensure reproducibility. Overall, although these publications are excellent resources for the analysis of quinine, they are not particularly written as method papers and are not readily followed by less-experienced personnel such as undergraduates. To fill this gap, this manuscript is designed to be a more complete reference guide for quinine analysis in tonic waters targeted toward both students and instructors primarily in the fields of analytical chemistry, physical chemistry, spectroscopy, and chemical education. In fact, this protocol is a result of systematic experiments performed by several groups of students for their senior capstone project, where a total of 13 students distributed in five groups (2 or 3 students each) took part in the experiments.

Fluorescence spectroscopy is a widely used analytical technique that has many practical applications due to its high analytical sensitivity [20,21]. For example, a spectrofluorometer is typically used for inorganic chemistry applications such as for the determination of chromium and manganese in steel or aluminum in alloys [22]. It is also commonly applied in the identification and quantification of organic compounds such as polycyclic aromatic hydrocarbons [23]. In principle, fluorescence is the emission of a molecule from its singlet excited electronic state to its ground state upon the absorption of UV or visible radiation [20,22]. Because of the direct relationship between the intensity of fluorescence and the concentration of an analyte in dilute solutions, fluorescence spectroscopy has been used to quantify quinine in tonic water samples. For example, several other studies have reported the use of custom-designed miniaturized fluorimeters for the detection and quantification of various compounds including quinine [24,25,26,27,28]. Compared to the previous studies, this work presents a systematically laid out protocol to measure the concentration of quinine in commercial tonic water samples using a spectrofluorometer while following strictly detailed step-by-step experimental and technical guidelines as provided in the Supplementary Information. In addition to determining the concentration of quinine in two tonic water brands (Canada Dry and Schweppes), the reproducibility and overall experimental precision of the method were assessed in this activity using five different sets of data collected independently. Overall, the level of quinine in tonic water was found to be within the acceptable range set by the FDA [3], and the method applied here was proven to be reliable given the high reproducibility of the data.

## 2. Materials and Equipment

Quinine sulfate dihydrate (QSD) was obtained from Mallinckrodt Pharmaceuticals, Cruiserath, Dublin, Ireland. The two one-liter tonic water bottles (Canada Dry and Schweppes) were purchased from commercial stores (e.g., Kroger, Richmond, VA, USA). Concentrated sulfuric acid (2.0 N, N stands for Normality, see Supplementary Information) and 18 MΩ deionized water were used to prepare the 0.05 M sulfuric acid solution (refer to Supplementary Information for more details on sample preparation and concentration calculations).

## 3. Hazards and Safety Precautions

Standard laboratory safety measures should be followed when handling hazardous chemicals. Quinine sulfate dihydrate (QSD) is a skin, eye, and respiratory irritant if inhaled. Precautions for QSD include wearing PPE, handling in a ventilated area, and avoiding inhalation of dust that can form.

## 4. Experimental Procedure

### 4.1. Sample Preparation

A 0.05 M sulfuric acid solution was prepared from the 2.0 N concentrated sulfuric acid solution and diluted with 18 MΩ deionized water (see the Chemicals and Samples section in the Supplementary Information). The prepared 0.05 M sulfuric acid solution was used as the diluent for all the subsequent standards and samples. A 1000 ppm quinine stock solution was prepared by weighing ~121 mg of QSD (structure and molecular weight information can be found in the Supplementary Information) and transferring it to a 100 mL volumetric flask, followed by dilution with 0.05 sulfuric acid. A 100 ppm quinine standard solution was prepared from the 1000 ppm solution, followed by further dilution to a 1 ppm quinine standard solution. Five calibration standards (0.2–5.0 ppm) were also prepared from the 100 ppm quinine standard in 25 mL volumetric flasks (see Table S1). The commercial quinine tonic water samples were prepared by delivering 2 mL of the tonic water sample into a 50 mL volumetric flask and diluting it with 0.05 M sulfuric acid (more details can be found in the Supplementary Information).

### 4.2. Generating Excitation and Emission Spectra

Excitation and emission spectra were collected for the 1 ppm quinine standard solution using the scan mode of the Agilent Varian-Cary eclipse fluorescence spectrometer with preinstalled scan and advanced reads programs for spectra recording and analysis (check the Supplementary Information for more details). Briefly, a quartz cuvette was filled with the 1 ppm solution and placed into the sample compartment of the spectrometer for analysis. To collect the excitation spectrum, the excitation monochromator was scanned over a wavelength range of 190–440 nm, while the emission monochromator was set to 450 nm. Similarly, two emission spectra were obtained by scanning the emission monochromator from 360 to 600 nm and setting the excitation either to 250 nm or 350 nm. For each spectrum, the slit widths of the monochromators were set to 5.0 nm and the photomultiplier tube (PMT) detector voltage was set to 600 V (Table S2).

### 4.3. Photomultiplier Tube (PMT) Voltage and Emission Intensity

The effect of PMT voltage on fluorescence intensity was assessed using the 1 ppm quinine solution and the advanced reads mode of the instrument software (refer to the Supplementary Information for more details). Briefly, the fluorescence intensity was measured as a function of PMT voltage, which varied from 400 to 725 V. The excitation and emission monochromators were set to 350 nm and 450 nm, respectively, and the slit widths were held constant at 5.0 nm.

### 4.4. Monochromator Slit Width and Emission Intensity

The effect of the excitation and emission monochromator slit widths on fluorescence intensity were independently analyzed using the 1 ppm quinine standard solution and the advanced reads mode of the instrument (see the PMT and Slit Width section in the Supplementary Information). The first part consisted of varying the excitation slit width from 1.5 to 20 nm while keeping the emission slit width at 2.5 nm, and the second part consisted of varying the emission slit width (1.5–20 nm) and maintaining the excitation slit at 2.5 nm. In both parts of the experiment, the excitation and emission monochromator wavelengths were set to 250 nm and 350 nm, respectively, and the PMT voltage was set to 600 V.

### 4.5. Statistical Analysis

All the experiments described here were performed by 5 groups of individuals (2–3 students each group). Each group provided one set of data for all experiments, and the mean value and standard deviation (S.D., shown as error bars) were calculated from five data sets (*n* = 5). All calculations were performed in Excel. It is important to note that the data were collected over the course of about 5 weeks (one group experimented per week); therefore, slight distribution observed in the datasets may be attributed to variations in instrument performance and/or sample preparation by different groups.

## 5. Results and Discussion

**Figure 1:**
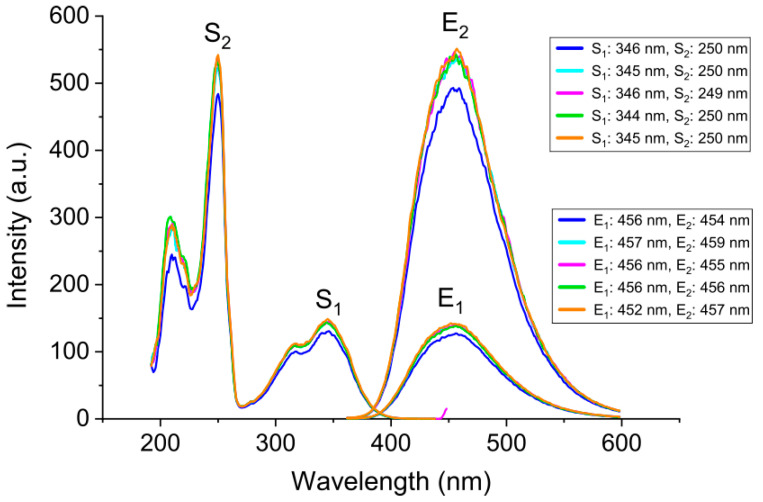
Excitation and emission spectra of the 1 ppm quinine standard solution. The spectra collected, one excitation and two emissions, are color-coded to designate the different data sets. Spectra were obtained with monochromator slit widths of 5.0 nm and a PMT voltage of 600 V. The λ_max_ values for the first excited singlet state (S_1_), second excited singlet state (S_2_), first emission (E_1_), and second emission (E_2_) are listed. Five replicates were recorded for excitation and emission spectra (*n* = 5). Spectra were collected at room temperature.

The first part of the experimental protocol consisted of a set of experiments performed to illustrate the effects of different instrumental parameters on the intensity of fluorescence emission (see the Supplementary Information for a step-by-step experimental protocol). Through these sets of experiments, the reproducibility of the instrument was also investigated across five different data sets collected independently. First, prior to recording fluorescence emission, excitation and emission spectra for the 1 ppm quinine solution were collected to select the wavelengths (λ_max_) for the subsequent experiments. The spectra from all five independent data sets are shown in Figure 1.

As depicted in Figure 1, the λ_max_ values did not vary significantly for the S_1_ and S_2_ peaks in the excitation spectra across the five sets of experiments. The mean wavelengths and standard deviations for S_1_ and S_2_ were 345 ± 1 nm and 250 ± 1 nm, respectively. In addition, the emission wavelengths were similar to one another (455 ± 2 nm for E_1_ and 456 ± 2 nm for E_2_). The observed λ_max_ values from the absorption and emission spectra of quinine are commensurate with the previously reported wavelengths of 347 nm and 452 nm for S_1_ and E, respectively [18]. The slightly higher standard deviation for the emission spectra is expected because of the different ways an excited electron can dissipate its energy within an electronic state (i.e., the presence of vibrational states within the ground electronic state, resulting in different emission wavelengths) [8,10]. Furthermore, variations could be due to instrument fluctuations or less successful nonradiative processes resulting in slightly different emission wavelengths. Unlike the λ_max_ values, the maximum intensity values for each listed wavelength were found to be less consistent. The mean maximum intensity values for the S_1_, S_2_, and E_1_ were 138 ± 13, 506 ± 50, and 133 ± 12, respectively. E_2_ (emission from excitation at 250 nm) was excluded from this analysis as it is not the λ_em_ chosen for the following experiments; thus, its intensity fluctuations are not as relevant and do not factor into the variations seen in the calculated quinine levels. As can be seen from the standard deviations of intensity, the maximum intensity values varied significantly between the five groups. Variations in fluorescence intensity are likely due to human errors (e.g., errors in weighing, pipetting, etc.) which can vary slightly from one group to another, resulting in different prepared concentrations of quinine. Moreover, quinine stock solutions had to be freshly prepared several times during the experiments, which might have resulted in slight differences in the final quinine concentrations. Notably, solutions at high concentrations can have an internal screening effect, leading to lower fluorescence intensities than expected.

**Figure 2:**
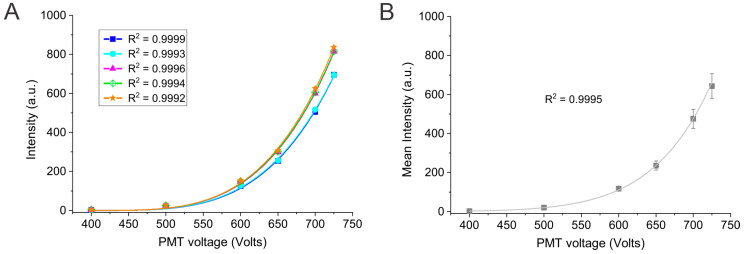
(**A**) Plot of fluorescence intensity vs. PMT voltage for five different data sets. Data points were best fit with the power function (second order), and the corresponding correlation coefficient R^2^ values are provided. (**B**) Plot of the mean fluorescence intensity and PMT voltage. The mean intensity was calculated by averaging out the five intensity values at each voltage, and the corresponding standard deviation is represented by the error bars (*n* = 5). The correlation coefficient for the best-fit line is shown on the plot.

The next portion of this study comprised varying the PMT voltage and measuring the fluorescence intensity. Intensities were measured at PMT voltages of 400–850 V using the 1 ppm quinine standard solution. The rationale behind this experiment is to examine the effect of the PMT voltage on the sensitivity of the instrument. Figure 2 illustrates the relationship between the PMT voltage and the measured intensity. It is evident from the plot in Figure 2A that the measured fluorescence intensity increased nonlinearly with increasing PMT voltage. This is expected because according to the photoelectric effect, as the PMT voltage increases, the voltage difference between the dynodes (i.e., electrodes) increases and, thus, the signal is exponentially amplified [29]. The correlation coefficient values retrieved from the best-fit curves for each group indicate a good correlation between the fluorescence intensity and PMT voltage. However, it is important to note that the data points corresponding to 800–850 V were omitted from the analysis as the fluorescence signal was saturated at high PMT voltages (the full curve including the signal saturation is provided in Figure S1).

Further examination of the reproducibility of fluorescence intensity at each PMT voltage revealed more variations at higher PMT voltages, which is obvious from the larger error bars (standard deviations) in Figure 2B. This observation is likely due to hitting the near-saturation limit of the PMT detector at higher voltages, which could affect the detection response and the signal reading. Nonetheless, the correlation coefficient obtained for the plot of mean fluorescence intensity and PMT voltage is comparable to the correlation coefficients obtained by the individual groups. Again, this illustrates a good correlation between the measured fluorescence intensity and PMT voltage. Overall, the data is more or less consistent at low-mid PMT voltages, which demonstrates good reproducibility of the data and a uniform instrument response.

**Figure 3:**
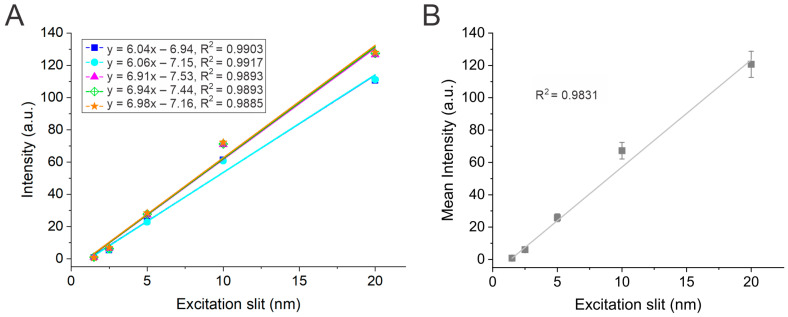
(**A**) Plot of fluorescence intensity vs. excitation monochromator slit width for the five groups. The linear best-fit lines, linear regression equations, and correlation coefficients are displayed for each group. (**B**) Plot of the mean fluorescence intensity as a function of excitation slit width. Error bars represent standard deviations from five datasets (*n* = 5). Data were collected at a PMT voltage of 600 V with an emission monochromator slit width of 5 nm.

Using the same approach, the 1 ppm quinine standard solution was also used to measure the fluorescence intensity while systematically varying excitation monochromator slit width. The intensity was measured at the slit widths of 1.5, 2.5, 5, 10, and 20 nm. Figure 3 shows the change in fluorescence intensity as a function of excitation monochromator slit width. As shown by the plot, the intensity increased linearly due to increasing excitation slit width. This direct linear relationship between intensity and excitation slit is illustrated by Equation (1) [29], where *I_F_*_(*λ*)_ is emission intensity, *P_o_* is the power of the incident radiation, $E_{\left(\lambda excit\right)}$ is the molar absorptivity at the excitation wavelength, *c* is the concentration, *l* is the pathlength, *Q_F_* is the quantum yield, and *k* is the ratio of absorbed to emitted photons.

(1) $$I_{F\left(\lambda \right)}=P_{o}\left(2.303E_{\left(\lambda excit\right)}cl\right)Q_{F}k$$

The rationale is that increasing the excitation slit width increases the amount of incident light and, thus, the number of photons that can be absorbed by the sample. This increases *k*, i.e., the ratio of absorbed to emitted photons, which increases the fluorescence intensity. As Equation (1) shows, the fluorescence intensity and ratio *k* are directly proportional, indicating a direct and linear relationship, provided other variables are constant. Variations in the measured intensity were identified among the independent data sets, particularly at larger slit widths (Figure 3B). Based on Equation (1), variations could be due to differences in the quinine standard concentration, which are more prominent at larger slit widths (i.e., higher *k*), or due to instrument fluctuations in the power of the incident radiation ($P_{o})$. It is important to note that changing the excitation monochromator slit width should not, in principle, affect the emission wavelength resolution, provided that the emission monochromator slit width is held constant.

**Figure 4:**
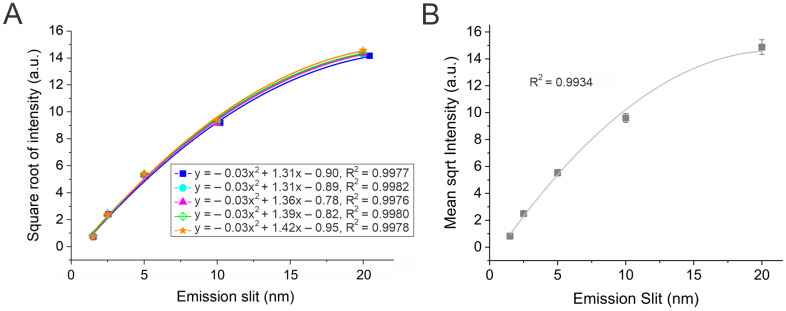
(**A**) Plot of the square root of fluorescence intensity vs. emission monochromator slit width for the five groups. The polynomial best-fit lines, quadratic equations, and correlation coefficients are displayed for each group. (**B**) Plot of the mean square root of intensity as a function of emission slit width. Error bars denote standard deviations from five datasets (*n* = 5). Data were collected at a PMT voltage of 600 V with an excitation monochromator slit width of 5 nm.

The last portion relating to instrumental parameters involved recording the fluorescence intensity at different emission monochromator slit widths. As in the excitation slit scenario, increasing the emission monochromator slit width is expected to increase the fluorescence intensity. This is due to the increased amount of emitted light permitted to reach the PMT detector. However, as shown in Figure 4A, which depicts the plot of the square root of intensity against emission slit width, the relationship was best represented by a quadratic (polynomial) rather than a linear function. This implies that the fluorescence signal is nonlinearly affected by the emission monochromator slit width.

Hence, we plotted the square root of the intensity as a function of the emission slit to highlight the difference in the instrument response to variations in excitation and emission slits. Unlike Figure 3, minimal variation in the measured intensity was noted between the groups, as shown in Figure 4B. This could be due to a smaller number of instrumental variables that contribute to variations in emission intensity given a constant excitation slit width. However, unlike excitation, increasing the emission monochromator slit width reduced the emission wavelength resolution.

After evaluating the instrument response and validating the reproducibility of the data, the concentration of quinine in commercial tonic water samples was determined and the results from the five groups were compiled and analyzed (check the External Calibration Standards section in the Supplementary Information). To determine the unknown concentration of quinine, five calibration standards with known concentrations of quinine were prepared and their fluorescence intensity was sequentially measured starting with the lowest concentration (refer to Table S3 for instrumental settings).

**Figure 5:**
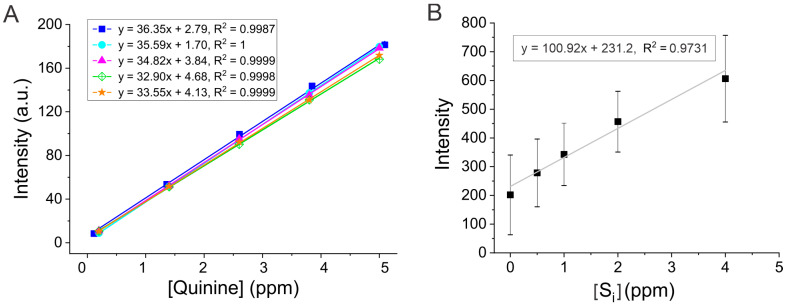
Calibration curves prepared from the quinine standard solutions using the external (**A**) and the internal (**B**) standard addition methods. The linear best-fit lines, linear regression equations, and correlation coefficients are displayed for each group in panel A (*n* = 5). Data were collected at excitation and emission wavelengths of 350 nm and 450 nm, respectively, with a PMT voltage of 600 V and slit widths set to 5 nm each. The Canada Dry tonic water sample was used for the internal standard (S_i_) method in panel B, and the fluorescence intensity is an average of two replicates (*n* =2). Standard deviations are represented by the error bars.

Figure 5 depicts two plots of fluorescence intensity as a function of quinine standard concentration in ppm. The resulting calibration curves demonstrated good linearity and high correlation coefficient values. Figure 5A shows the overlay of five calibration curves using the external standard method. A slight deviation in the measured intensity can be seen at higher quinine concentrations, which could be due to human error while preparing the standards or instrumental errors as described earlier. To cross-compare, we included another set of calibration data collected by an independent group of individuals shown in the Supplementary Information (Figure S2). Comparing the two sets of calibration curves, we can conclude that the data are reproducible with a good linearity (R^2^ > 0.99). The slight differences among experiments can be attributed to variations in sample preparations.

Using the linear regression equations determined from the calibration curves, the concentration of quinine in the tonic water samples (Canada Dry and Schweppes) was calculated. For the external standard method, the individual results from the five groups are shown in Table 1 along with the mean quinine concentration and standard deviations. As described in the experimental section and SI, the tonic water samples were diluted before recording the fluorescence measurements, and, thus, there are two quinine concentrations denoted as diluted and undiluted, as shown in Table 1. The undiluted concentration represents the actual quinine concentration in the tonic water sample considering the dilution factor (25-fold). As illustrated in Table 1, there is some variation in the calculated quinine concentrations among the five groups, especially for the Canada Dry tonic water sample.

This is to be expected considering that the measurements were taken at different times/days and the possibility of minor variations in sample preparation. However, the results are consistent as the standard deviations are relatively small. The mean concentrations of the two tonic water samples were determined to be approximately 65 ppm for both samples, which is slightly higher than the average concentration range of quinine in commercial tonic water (~25–60 ppm) [4]. However, the quinine concentration of ~65 ppm is well below the FDA requirements of 83 ppm or less for quinine [3,24]. Fitting the calibration data also allowed us to estimate the limit of detection (LOD) of quinine, which was 0.2 ppm.

In addition, the quinine concentration was also determined using an internal standard approach as a complementary method (Figure 5B). The standard addition allows for the measuring of fluorescence in the presence of any interferences caused by the sample matrix. In this experiment, the Canada Dry sample was used as an example and the quinine quantity was determined in the sample (using two replicates) and cross-compared with the result from the external standard method. Briefly, the quinine concentration was calculated using the extrapolation of data, in which the x-intercept represents the concentration of the unknown quinine sample [Q]_x_ (Figure 5B). Given the 25-fold dilution, the extrapolated concentration of quinine was multiplied by the dilution factor to calculate the actual (undiluted) concentration, which was found to be ~57 ± 26 ppm. This result is comparable to the concentration in the Canada Dry sample (~65 ± 4 ppm) determined using the external calibration method (Figure 5A). The lower quinine concentration and larger error determined by the internal standard method may be attributed to the matrix effect. Further, the day-to-day reproducibility of the method is inclusive, given that the data were collected on different days by independent groups of students. The consistent fluorescence data and calculated quinine levels confirm the reproducibility of the method over the course of days/weeks. Taken together, the quinine concentration is consistent with the concentration ranges of 57–80 ppm, 48–67 ppm, and 62–67 ppm in different tonic water samples reported previously using a PerkinElmer FL6500 fluorescence spectrometer, a LS-50B luminescence spectrometer, and a reverse-phase HPLC method, respectively [2,16,18].

Furthermore, it is critical to probe the effect of interferences such as pH, food additives, and/or artificial sweeteners, including citric acid and sugars. Previous studies investigated the effect of chloride ions and artificial additives (e.g., glucose) as major interferences and found that chloride ions can cause fluorescence quenching; however, no appreciable interference from sugar additives was noted [2,4]. More relevantly, maintaining an acidic environment is important when using the approach presented here; hence, we sought to check the effect of pH on the quinine fluorescence intensity, wherein we measured fluorescence emission as a function of pH (Figure S3). The detailed procedure is outlined in the Methods section of the Supplementary Information. Briefly, sodium phosphate buffers were made with a pH range of ~6.0–7.9 to which a constant amount of quinine (10 ppm) was added before measuring the fluorescence. The results showed that, as the pH increases, the fluorescence intensity slightly decreases, possibly due to some level of the deprotonation of quinine (pKa 8.4) when increasing the pH [30]. Nonetheless, it is important to note that all the fluorescence experiments in this activity were performed in an acidic solution (0.05 M H_2_SO_4_) that should keep quinine protonated and fluorescent.

## 6. Conclusions

This work highlights the fundamentals and principles of fluorescence spectroscopy and its application in the detection and quantitation of analytes. Using this technique, the effects of instrument parameters on fluorescence intensity were investigated and the amounts of quinine in commercial tonic water samples were successfully determined. The results from the five different data sets demonstrated the relationships between instrument parameters and fluorescence intensity, showing an overall good reproducibility among the data. Additionally, the calibration curves using quinine standards and the quantitation of the quinine levels in tonic water samples produced consistent results, with calculated concentrations that are well below the maximum concentration limit of quinine that is FDA-approved. Overall, besides the simple and instructive protocol that we developed for quinine, this facile and low-cost spectroscopic technique may be adopted to determine the amount of other relevant drugs and food additives. The presented method and detailed experimental protocol can be further implemented in undergraduate teaching labs and in chemical education.

## Figures and Tables

**Table 1:** Measured intensity and calculated concentrations (diluted and undiluted) of quinine in the two commercial tonic water samples. Results represent the mean intensity, mean diluted, and undiluted concentration of quinine with standard deviations of the five data sets (*n* = 5). Data were acquired at excitation and emission wavelengths of 350 nm and 450 nm, respectively, and a PMT voltage of 600 V with slit widths set to 5 nm each.

Student Group	Tonic Water	Intensity at 450 nm	Diluted Quinine (ppm)	Undiluted Quinine (ppm)
**1**	Sample 1 (Canada Dry)	92.59 ± 1.21	2.47	61.76
	Sample 2 (Schweppes)	90.72 ± 2.10	2.42	60.48
**2**	Sample 1 (Canada Dry)	87.43 ± 0.15	2.41	60.23
	Sample 2 (Schweppes)	94.96 ± 1.61	2.62	65.52
**3**	Sample 1 (Canada Dry)	93.91 ± 0.66	2.59	64.67
	Sample 2 (Schweppes)	91.85 ± 1.99	2.53	63.19
**4**	Sample 1 (Canada Dry)	95.19 ± 1.02	2.75	68.78
	Sample 2 (Schweppes)	91.66 ± 0.33	2.64	66.09
**5**	Sample 1 (Canada Dry)	94.80 ± 0.78	2.70	67.57
	Sample 2 (Schweppes)	94.31 ± 1.35	2.69	67.21
**Results:**	Sample 1 (Canada Dry)	92.78 ± 3.16	2.58 ± 0.15	64.60 ± 3.66
	Sample 2 (Schweppes)	92.70 ± 1.83	2.58 ± 0.11	64.50 ± 2.68

## Data Availability

The data is available upon reasonable request from the authors.

## Supplementary Materials

The following supporting information can be downloaded at: www.mdpi.com/article/10.3390/mps8010005/s1, Figure S1: Plot of fluorescence intensity vs PMT voltage. Data were collected at excitation and emission wavelengths of 350 nm and 450 nm and slit widths of 5 nm. The signal saturates at PMT voltage of ~800 V and above; Figure S2: Calibration curves prepared from the quinine standard solutions using the external standard method. The linear best-fit lines, linear regression equations, and correlation coefficients are displayed for each group. Data were collected at excitation and emission wavelengths of 350 nm and 450 nm, respectively, with a PMT voltage of 600 V and slit widths set to 5 nm each. The data presented were collected by a different group of individuals to supplement the calibration curves in Figure 5A (main text); Figure S3: pH dependence of quinine fluorescence intensity. Data were collected at excitation and emission wavelengths of 350 nm and 450 nm, respectively, with a PMT voltage of 600 V and slit widths set to 5 nm each. The concentration of quinine was 10 ppm in the buffer systems and the pH was measured after the addition of all components using a pH meter. Error bars represent the standard deviations from three replicates of intensity measurements; Table S1: External standard quinine solutions for the calibration curve including their respective concentration and volume transferred; Table S2: Internal standard quinine solutions with their respective concentrations and volume added; Table S3: Measured amounts of the phosphate buffer components and the respective final pH of the buffer solutions for the pH dependence test; Table S4: Instrumental parameters and settings for generating excitation and emission spectra; Table S5: Instrument settings to generate intensity measurements for the quinine standards and tonic water samples.
