# Supplementary material for: Fluorescence Analysis of Quinine in Commercial Tonic Waters
Source: Methods Protoc. 2025 Jan 6;8(1):5. doi: 10.3390/mps8010005 (PMC11755655; doi:10.3390/mps8010005)
Supplement: Supplementary file 1 [file mps-08-00005-s001.zip › mps-3322083-supplementary.pdf]

Supporting Information

Fluorescence Analysis of Quinine in Commercial Tonic Waters

Artturi Harcher, Connor Ricard, Danielle Connolly, Isabel Gibbs, Jarve Shaw, Jillian Butler, Julia Perschbacher, Lindsay Replogle, Michaela Eide, Morgan Grissom, Oliver O'Neal, Quan Nguyen, Van Hac Nguyen, Michael Hunnicutt, Roaa Mahmoud † and Soma Dhakal *

Department of Chemistry, Virginia Commonwealth University, 1001 West Main Street, Richmond, VA 23284
† Graduate Teaching Assistant
* Correspondence: sndhakal@vcu.edu

Materials and Methods

Chemicals and Samples

Preparation of H_2SO_4 solution. A total of 2.0 N H_2SO_4 (stock solution) can be prepared from a concentrated 18 M sulfuric acid solution and diluted with 18 MΩ deionized water. The prepared 2.0 N sulfuric acid is used to prepare the diluent 0.05 M sulfuric acid to dilute the quinine standards and samples with. This was achieved by transferring 25 mL of the 2.0 N H_2SO_4 solution into a 500 mL volumetric flask and diluting to volume with 18 MΩ deionized water. Caution must be taken when handling concentrated sulfuric acid as it can cause severe skin, eye, and respiratory irritations. Therefore, it is advisable that the sulfuric acid solutions are kept in a ventilated space such as a fume hood with proper PPE to limit exposure to the hazards posed by sulfuric acid and to minimize health risks as much as possible.

Note: Concentration expressed as normality (N) = molarity (M) × (# of equivalent units of H^+ ion generated per mole).

Preparation of standard quinine stock solutions. Quinine Sulfate Dihydrate (QSD), purchased from Mallinckrodt Pharmaceuticals, is used to prepare the standard quinine stock solutions (the chemical formula, structure, and molecular weight of QSD are provided below for reference). Specifically, to make a 1000 ppm quinine stock solution, 0.1207 g of QSD was weighed on a measuring balance, the amount was transferred into a 100 mL volumetric flask, and it was diluted with 0.05 M sulfuric acid to the 100 mL mark. A secondary stock solution of 100 ppm quinine was also prepared from the 1000 ppm quinine solution by delivering 10 mL using a volumetric pipet into a 100 mL volumetric flask and diluting with 0.05 M sulfuric acid.

Quinine Sulfate Dihydrate (MW = 782.94 g/mole) formula: $(C_{20}H_{24}N_2O_2)_2 \cdot H_2SO_4\ 2H_2O$

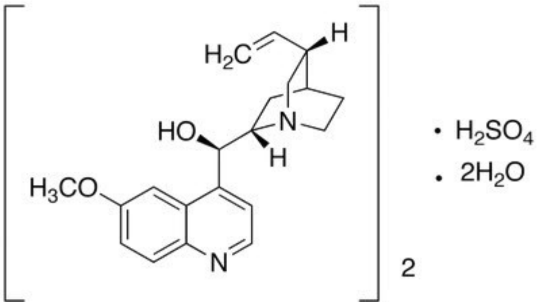

The quinine structure is taken from Ref 1.

External standard method. To create the external calibration curves for quinine (see Fig. 5A in the main text), five external standard quinine solutions were prepared from the 100 ppm quinine stock using 25 mL volumetric flasks and calibrated micropipettes with volume ranges of 10-100 µL and 100-1000 µL (five replicate sets of standards used). The five external standards with their corresponding concentrations were prepared as follows.

Table S1. External standard quinine solutions for the calibration curve including their respective concentration and volume transferred.

Quinine Standard (ppm)	Volume of Quinine Stock (100ppm) needed (μL)	Final Volume (mL)
0.2	50	25
1.4	350	25
2.6	650	25
3.8	950	25
5.0	1250	25

An additional standard of 1 ppm quinine was also made in a 25 mL flask to acquire excitation andemission spectra (Fig. 1 main text) and test the accuracy of the instrumental parameters such as PMT voltage, excitation, and emission monochromator slit widths (see Figs 2-4 main text). All standards were diluted to the 25 mL mark with the 0.05M sulfuric acid diluent.

Internal standard method. A set of five quinine standards using the internal standard method were prepared to compare quinine concentration outcomes with the external standard method described above. Briefly, five 25 mL volumetric flasks were used, to which 1 mL of the Canada Dry tonic water sample was added. Internal quinine standard was added to a final concentration range of 0.5-4 ppm only to four flasks using the 100 ppm quinine stock and calibrated micropipettes with volume ranges of 100-1000 μL, as shown in the table below. All solutions were diluted using the 0.05M sulfuric acid diluent. Two replicates of internal standards were used to create the internal standard calibration curve shown in Figure 5B (main text).

Table S2. Internal standard quinine solutions with their respective concentrations and volume added.

Solution	Volume of internal quinine standard (μL)	Internal standard concentration (ppm)	Volume of Canada Dry sample (μL)
1	0	0	1000
2	125	0.5	
3	250	1	
4	500	2	
5	1000	4	

Tonic water samples. Two commercial tonic water samples were used in this experiment, specifically, Canada Dry and Schweppes tonic water bottles of 1 liter each. However, other types of tonic water such as Polar, Kroger, or Harris Teeter can also be used. The purpose of using two samples is to compare the quinine levels across different tonic water brands and to determine whether they are within the allowable concentration range of quinine in beverages set by the FDA (≤ 83 ppm [Refs 2,3]; see Table 1 in the main text). The samples were diluted by taking 2 mL from the tonic water using volumetric pipets and transferring it into 50 mL volumetric flasks, followed by dilution with 0.05 M sulfuric acid. The concentration of quinine in the original sample was, therefore, calculated using the fluorescence emission readings of the diluted samples while taking into consideration the dilution factor that was applied (25-fold dilution, 2 mL of sample aliquoted into a 50 mL flask).

pH dependence of quinine fluorescence. The effect of pH on the fluorescence intensity of quinine was examined by preparing four phosphate buffer solutions with pH values ranging from ~6.0 to 7.9. Solutions (10 mL each) were prepared using appropriate amounts (g) of sodium phosphate dibasic anhydrous (Na_2HPO_4) and sodium phosphate monobasic anhydrous (NaH_2PO_4) as outlined below (Table S3). Quinine was added to each buffer solution to a final concentration of 10 ppm using the 100 ppm quinine stock. The solutions were diluted with deionized water to a final volume of 10 mL, and the pH was adjusted using potassium hydroxide (KOH) when necessary. The final pH was measured using a pH meter.

Table S3. Measured amounts of the phosphate buffer components and the respective final pH of the buffer solutions for the pH dependence test.

**Target pH**	**Phosphate buffer**	**Amount (g)**	**Quinine (μL)**	**# of KOH drops**	**Final pH**
	Na_2HPO_4	0.0196			
**6.0**	NaH_2PO_4	0.1297		1	5.95
	Na_2HPO_4	0.0511			
**6.5**	NaH_2PO_4	0.0779		1	6.56
	Na_2HPO_4	0.0823			
**7.0**	NaH_2PO_4	0.0513	1000	1	6.94
	Na_2HPO_4	0.1145			
**7.5**	NaH_2PO_4	0.0252		2	7.85

Instrumentation and Common Accessories

- Agilent Varian-Cary Eclipse Fluorescence Spectrometer
- Quartz sample cuvettes (1 cm x 1 cm) for experiments using 1 ppm quinine solution
- Plastic sample cuvettes (1 cm x 1 cm) for quantitative analysis (i.e., standards and samples)
- Volumetric flasks containing the 1 ppm solution, the standards, and the samples (placed in an appropriate beaker to avoid spillage)
- Wash bottle filled with DI water
- Waste beaker (1000 mL)
- Box of Chemwipes
- Disposable Pasteur glass pipets

Step-By-Step Standard Operating Procedure (SOP): Varian-Cary Eclipse Fluorescence Spectrometer

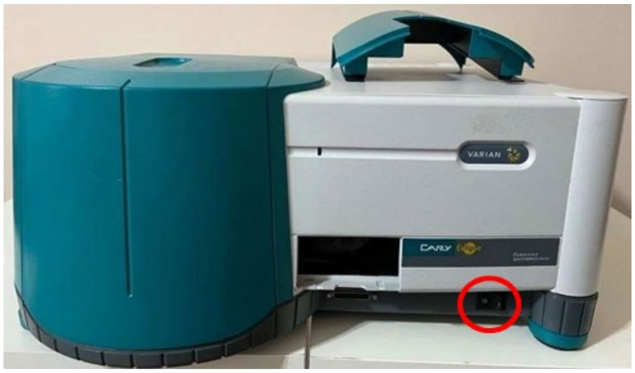

Step 1: Turn on the spectrometer (shown above). The power switch is located on the bottom-right corner of the instrument. Flip the switch (circled in red) from the [O] position to the [| |] position.

Note: Turn on the instrument at least 30 min before taking any measurements to allow the lamp enough time to warm up.

Step 2: Select the instrument mode of operation (i.e. Scan or Advanced Reads modes). These two software programs are part of the Agilent CaryWinFLR fluorescence software package of the instrument. Locate the Scan and Advanced Reads icons shown below that will be used to control the instrument. The Scan mode is used to collect emission intensity as a function of excitation wavelength. The two types of spectra that can be collected are excitation and emission spectra. The Advanced Reads mode is used to collect the emission intensity generated at a specific wavelength.

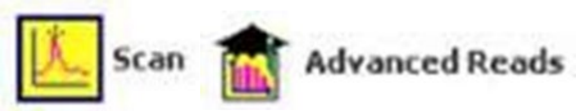

Step 3: Create a data file folder into which all data will be saved.

Generating Excitation and Emission Spectra

Step 1: Open the Scan mode program. This will be used to create excitation and emission spectra for 1 ppm quinine. Locate the Control Bar at the top-left corner of the Scan page. Move the mouse cursor over the <View> command and make sure the following features are enabled (√). See the image below.

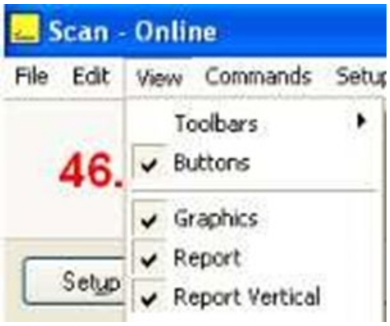

Step 2: Locate the <Set Up> icon just below the Control Bar. Click on the setup icon and the following tabs: **Cary**, **Options**, **Accessories**, **Reports**, and **Auto-Store** will appear at the top of the setup page (see Figure below). In the [CARY] tab, confirm that the instrument mode is set to <Fluorescence>. Update the Scan setup using the instrument settings listed in the table below.

Table S4. Instrumental parameters and settings for generating excitation and emission spectra.

Type of Spectrum	Excitation Monochromator (M1)	Emission Monochromator (M2)
Excitation	190 – 440 nm	450 nm
Emission	250 nm	360 – 600 nm
Emission	350 nm	360 -600 nm

For each spectrum, set M1 and M2 Slit Width = 5.0 nm and the PMT Detector Voltage = medium(600 V). Set the Scan Control = medium.

Step 3: Click the <OPTIONS> tab. Locate the Display Options section and check the <Overlay traces> box. Locate the Smoothing section and check the <Smoothing> box. Select the <Satvisky- Golay> Smoothing function and set <Factor> to 5. Locate the PMT Detector Voltage section and verify that it is set to medium (600 V).

Note: Skip the <ACCESSORIES> tab.

Step 4: Click the <REPORTS> tab. The Options and Peaks sections within the Reports tab should be modified to match the settings shown below with the exception of the Peak Threshold.

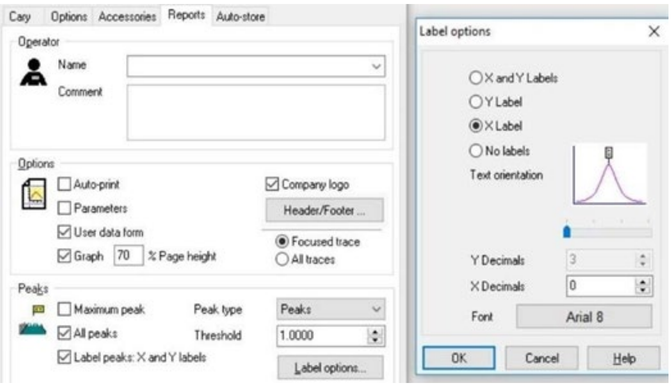

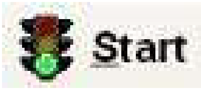

<u>Note</u>: Set Threshold to **50.000** for all experiments.

Click on the Label Options icon in the Peaks section and select <X label> only. Set <X Decimals> to 0. The setup is now complete. Click the <OK> icon at the bottom of the screen to return to the main SCAN screen.

<u>Step 5</u>: Locate a clean and dry quartz cuvette. Rinse the cuvette several times with the 1 ppm quinine solution, fill three-quarters of it with 1 ppm quinine, and wipe the outside with a Chemwipe to make sure the cell walls are clean prior to placing it in the sample cell compartment.

<u>Step 6</u>: Place the filled cuvette in the sample chamber and close the cover. Locate the <START> icon (shown above) at the top of the page and click to begin the Scan.

The [Sample Name] screen will appear. You may either click OK and leave it unnamed or revise the name to something more descriptive of the sample.

<u>Step 7</u>: Save your spectral data as described below (see the Saving Spectral Data section). You may erase existing spectra or intensity data from the computer screen by clicking the "Trash Can" icon 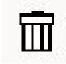 at the top of the Scan page. Repeat <u>Step 5</u> until all the spectra for the experiment have been collected.

Saving Spectral Data

Raw spectral data files are saved in the Spreadsheet Ascii (*.CSV) format. Saving data files in this format allows you to reconstruct the spectra using Excel. All the spectra visible on the screen will be saved in the data file.

1. Locate the Control Bar shown below at the top leftcorner of the SCAN screen.

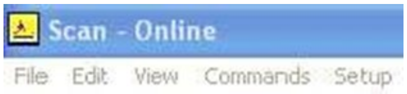

2. Place the mouse cursor over the <File> option and click on the <Save As> option. If you do notsee your folder, place the cursor in the <Look in:> area and select the folder you created.

3. Enter a file name for the data you are saving and set the file type to spreadsheet ASCII *.CSVfile format. Click <Save>.

Converting a CSV File to an Excel File

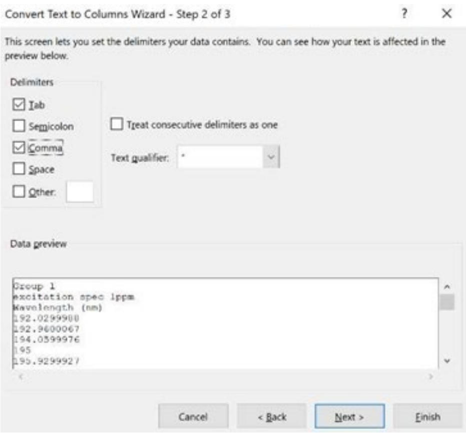

1. Open Excel and click on <File> then <Open>. Select <All Files> so you can see the CSV files in your folder.Select the CSV file and click <Open>.
2. Click on the first column. Click on <Data>, select <Textto Columns> in the Data Tools area, and select <Delimited> format. Next, select <Commas and Tabs>as delimiters to separate each data field. Click <Next>,select the <General> column format, and click <Finish>.The file should now have distinct columns.
3. Save the Excel file as a Workbook (.xlsx).

PMT and Slit Width Experiments

The 1 ppm quinine solution was used for all measurements. The measurements for both experiments were made using the **Advanced Reads** mode.

Step 1: Click on the <Windows> icon located in the lower-left corner of the DESKTOP screen. Locate the Advanced Reads program icon. Click on the icon to enable the Advanced Reads software.

Step 2: Locate the Control Bar at the top-left corner of the Advanced Reads page. Move the mouse cursor over the <View> command and make sure functions are enabled (√). See example below.

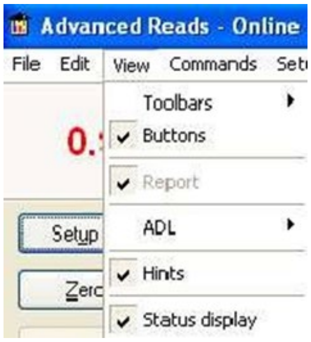

Step 3: Locate the <Set Up> icon just below the Control Bar. Click on the icon and six tabs will appear at the top of the setup page.

Step 4: Confirm the instrument mode is set to <Fluorescence> and set the <Averaging Time, Ave.time(s)> to 0.1000 sec. Refer to the instrumental parameters listed below to update the setup settings and collect data for experiment A.

A. Varying the PMT voltage; constant monochromator slit widths:

- Excitation Monochromator (M1): 350 nm, slits = 5.0 nm
- Emission Monochromator (M2): 450 nm, slits = 5.0 nm
- PMT Voltages (V): 400 (Low), 500, 600 (medium), 650, 700, 725, 800 (High)

Note: The instrument will read *Over Range* if the intensity value is greater than 1000. If this occurs,determine the highest PMT voltage to generate an on-scale intensity and include it in your data set.

Step 5: Click the <Samples> tab. Enter 1 for the number of samples. Click on the <Replicates> andenter 3 to make each measurement in triplicate. Click the <OK> icon.

Step 6: Place the filled cuvette in the sample chamber and close the cover. Locate the <START> icon at the top of the page and click to acquire intensity data.

Note: Record the mean intensity and % relative standard deviation values.

Step 7: Repeat Step 4 until you have entered all the settings listed above for the wavelength and PMT voltage and completed the data collection.

Step 8: Click the <Clear Report> icon to clear the screen.

To acquire data for experiments B and C, repeat steps 4-7 using the instrumental settings provided below.

B. Varying the emission monochromator slit width; constant excitation monochromator slit width:

- PMT voltage: 600 V (medium)
- Excitation Monochromator (M1): 350 nm, slit width = 2.5 nm
- Emission Monochromator (M2): 450 nm
- Emission Monochromator Slit Widths (M2): 1.5, 2.5, 5, 10, and 20 nm

IMPORTANT: The slit width should be reset prior to each measurement and the mean intensity value for each slit width setting should be recorded.

C. Varying the excitation monochromator slit width; constant emission monochromator slitwidth:

- PMT voltage: 600 V (medium)
- Emission Monochromator (M2): 450 nm, slit width = 2.5 nm
- Excitation Monochromator (M1): 350 nm
- Excitation Monochromator Slit Widths (M1): 1.5, 2.5, 5, 10, and 20 nm

IMPORTANT: The slit width should be reset prior to each measurement and the mean intensity value for each slit width setting should be recorded.

Note: At the end of the experiments, remove the cuvette from the sample compartment and rinse itwith DI water.

External Calibration Standards and Tonic Water Samples

In this part, the five standards (0.2 – 5 ppm) and the two tonic water samples (Canada Dry and Schweppes) are analyzed using the **Advanced Reads** mode of the instrument software. Unlike previous experiments, which used a quartz cuvette, in this experiment, plastic cuvettes are usedand can be arranged in two rows as specified.

First Row: External Standard Solutions (arranged from lowest to highest concentration).

Second Row: Diluted Tonic Water Samples.

Step 1: Locate the <Set Up> icon just below the Control Bar. Click the icon and six tabs will appear at the top of the setup page.

Step 2: Confirm the instrument mode is set to <Fluorescence> and set the <Averaging Time, Ave.time(s)> to 0.1000 sec. Use the wavelength and PMT settings shown in the table below (Table S5) to update the setup. All other settings are left in their default setting.

Table S5. Instrument settings to generate intensity measurements for the quinine standards and tonic water samples.

Type of Data	Excitation Monochromator (M1)	Emission Monochromator(M2)
Fluorescence Emission	350 nm	450 nm
Set M1 and M2 Slit Width = 5.0 and the PMT Detector Voltage = medium (600 V) for allmeasurements. Set the Scan Control = medium.		

Step 3: Click the <Samples> tab. The number of samples should be equal to the total number of calibration standards and tonic water samples to be analyzed (in this case, the number of samplesshould be 7).

Step 4: Click on the <Replicates> and enter <3> so each measurement is made in triplicate.

The Sample Name can be revised. For example: Standard Set—use the concentration (ppm) or volume of 100 ppm stock used to prepare the standard solution (i.e., 0.050 mL). Click the <OK> icon when finished.

Step 5: Rinse each cuvette with its respective solution that will go in it. Fill the cuvette ¾ full andwipe the cell with a Chemwipe to make sure the cell walls are clean before placing it into the cuvette holder.

Step 6: Locate the <START> icon at the top and click to begin data collection.

A new sub-screen will appear entitled **SAMPLE SELECTION**. Click the <OK> icon.

The **PRESENT SAMPLE** window shown below willappear. This is a signal to place the first sample into the cuvette holder and then close the sample compartment.

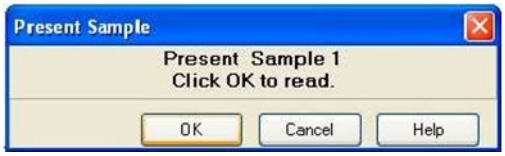

Step 7: Click the <OK> icon to begin data collection for this sample.

Note: The instrument will collect the data for sample 1 and then present to you the next sample (i.e.,sample 2 or whatever you named that sample). Before clicking the <OK> icon for sample 2, placethe cuvette for that sample into the compartment. Start the data collection.

Repeat Step 7 until all samples in the solution set have been analyzed.

Step 8: Click the <Print Report> icon and select a printer. Alternatively, data can be saved as a pdf version as described below or recorded (i.e., mean intensity and % relative standard deviation values for each sample) in your notebook.

Saving Data as a PDF File in the Advanced Reads Mode

A PDF version of the intensity values visible on the screen can be created.

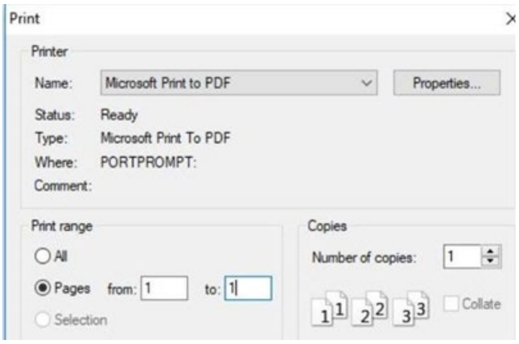

1. Locate and click on the <Print> icon in the bottom-left corner of the screen.

2. Use the drop-down menu in the <**Printer Name:**> field (see image above).
3. Select the **Microsoft Print to PDF** option.
4. Set <Copies> to 2 if working with a partner.
5. Click on the <Browse> icon and select your data folder. Enter a file name and select **PDFFile** in the <Save as File: > field.

Step 9: Once you have a copy of your data, click the <Clear Report> icon to clear the screen. Exit from the Advanced Reads software.

Step 10: Copy your data files from the folder to your USB. Turn off the instrument by toggling the power switch from the [I] position to the [O] position.

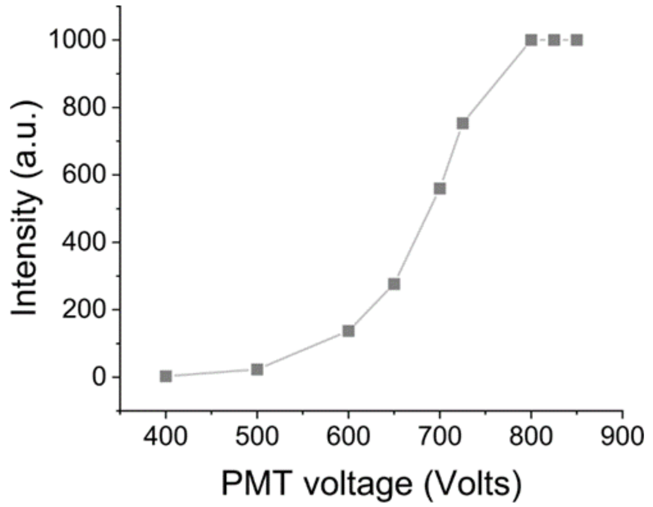

Figure S1. Plot of fluorescence intensity vs PMT voltage. Data were collected at excitation and emission wavelengths of 350 nm and 450 nm and slit widths of 5 nm. The signal saturates at PMT voltage of ~800 V and above.

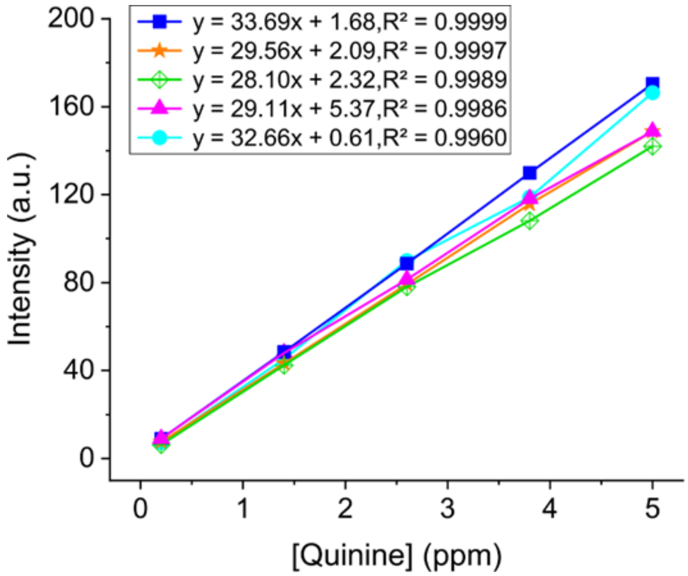

Figure S2. Calibration curves prepared from the quinine standard solutions using the external standard method. The linear best-fit lines, linear regression equations, and correlation coefficients are displayed for each group. Data were collected at excitation and emission wavelengths of 350 nm and 450 nm, respectively, with a PMT voltage of 600 V and slit widths set to 5 nm each. The data presented were collected by a different group of individuals to supplement the calibration curves in Figure 5A (main text).

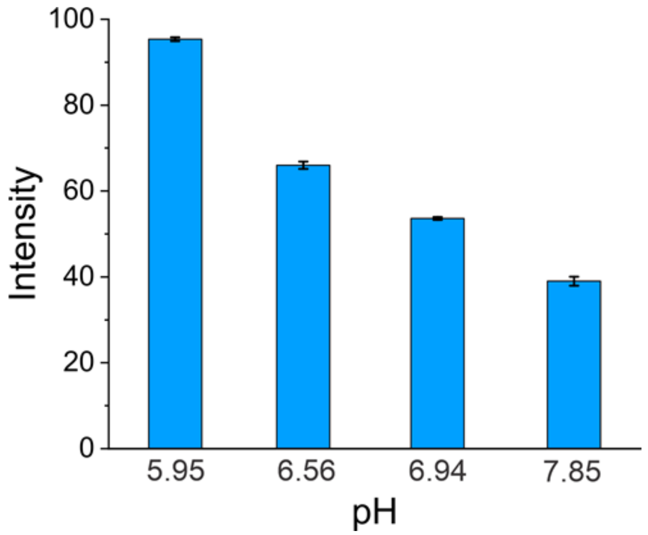

Figure S3. pH dependence of quinine fluorescence intensity. Data were collected at excitation and emission wavelengths of 350 nm and 450 nm, respectively, with a PMT voltage of 600 V and slit widths set to 5 nm each. The concentration of quinine was 10 ppm in the buffer systems and the pH was measured after the addition of all components using a pH meter. Error bars represent the standard deviations from three replicates of intensity measurements.

References

1. Millipore Sigma Quinine Sulfate Dihydrate. Sigma Aldrich. Available online: https://www.sigmaaldrich.com/US/en/substance/quininesulfate39147207671441 (Accessed on 7 May 2023).
2. U.S. Food and Drug Administration (FDA) Code of Federal Regulations - Title 21 - Food and Drugs; Sec.172.575. Available online: http://www.accessdata.fda.gov/scripts/cdrh/cfdocs/cfCFR/CFRSearch.cfm?fr= 172.575 (Accessed on 5 October 2024).
3. O'Reilly, J. E. Fluorescence Experiments with Quinine. *J. Chem. Educ.* **1975**, *52* (9), 610. DOI: 10.1021/ed052p610.

VCU Chemistry

Virginia Commonwealth University

Advanced Reads Report

Report Time : Tue 27 Sep 01:53:30 PM 2022

Software Version: 1.2(146)

Operator:

Instrument Parameters

Instrument	Cary
Eclipse Instrument Serial	Number
	EL000434
40 Data mode	Fluores-
cence	
Ex. Wavelength (nm)	350.00
Em. Wavelength (nm)	450.00
Ex. Slit (nm)	5
Em. Slit (nm)	5
Ave Time (sec)	0.1000
Excitation filter	Auto
Emission filter	Open
PMT Voltage (V)	600
Replicates	3
Sample averaging	OFFComments:

Analysis

Collection time 9/27/2022 1:53:31 PM

Sample	F	Mean	SD	%RSD	Readings
Standard 1					8.474
					8.710
					7.927
		8.3702	0.4017	4.80	
Standard 2					53.846

				54.365
				52.429
	53.5469	1.0021	1.87	
Standard 3				101.111
				98.246
				99.035
	99.4636	1.4800	1.49	
Standard 4				145.874
				142.935
				142.432
	143.7469	1.8592	1.29	
Standard 5				183.644
				180.084
				180.357
	181.3618	1.9813	1.09	
Unknown 1 C				91.500
				93.898
				92.364
	92.5874	1.2147	1.31	
Unknown 2 S				92.935
				90.475
				88.761
	90.7236	2.0980	2.31	
